# DNA Methylation Profiles of Purified Cell Types in Bronchoalveolar Lavage: Applications for Mixed Cell Paediatric Pulmonary Studies

**DOI:** 10.3389/fimmu.2021.788705

**Published:** 2021-12-22

**Authors:** Shivanthan Shanthikumar, Melanie R. Neeland, Richard Saffery, Sarath C. Ranganathan, Alicia Oshlack, Jovana Maksimovic

**Affiliations:** ^1^ Respiratory and Sleep Medicine, Royal Children’s Hospital, Parkville, VIC, Australia; ^2^ Department of Paediatrics, University of Melbourne, Parkville, VIC, Australia; ^3^ Respiratory Diseases, Murdoch Children’s Research Institute, Parkville, VIC, Australia; ^4^ Molecular Immunity, Murdoch Children’s Research Institute, Parkville, VIC, Australia; ^5^ Computational Biology Program, Peter MacCallum Cancer Centre, Parkville, VIC, Australia; ^6^ Sir Peter MacCallum Department of Oncology, University of Melbourne, Parkville, VIC, Australia; ^7^ School of BioScience, University of Melbourne, Parkville, VIC, Australia

**Keywords:** DNA methylation, bronchoalveolar lavage, paediatrics, cystic fibrosis, pulmonary disease, epigenetics

## Abstract

In epigenome-wide association studies analysing DNA methylation from samples containing multiple cell types, it is essential to adjust the analysis for cell type composition. One well established strategy for achieving this is reference-based cell type deconvolution, which relies on knowledge of the DNA methylation profiles of purified constituent cell types. These are then used to estimate the cell type proportions of each sample, which can then be incorporated to adjust the association analysis. Bronchoalveolar lavage is commonly used to sample the lung in clinical practice and contains a mixture of different cell types that can vary in proportion across samples, affecting the overall methylation profile. A current barrier to the use of bronchoalveolar lavage in DNA methylation-based research is the lack of reference DNA methylation profiles for each of the constituent cell types, thus making reference-based cell composition estimation difficult. Herein, we use bronchoalveolar lavage samples collected from children with cystic fibrosis to define DNA methylation profiles for the four most common and clinically relevant cell types: alveolar macrophages, granulocytes, lymphocytes and alveolar epithelial cells. We then demonstrate the use of these methylation profiles in conjunction with an established reference-based methylation deconvolution method to estimate the cell type composition of two different tissue types; a publicly available dataset derived from artificial blood-based cell mixtures and further bronchoalveolar lavage samples. The reference DNA methylation profiles developed in this work can be used for future reference-based cell type composition estimation of bronchoalveolar lavage. This will facilitate the use of this tissue in studies examining the role of DNA methylation in lung health and disease.

## Introduction

DNA methylation (DNAm) is the most widely studied epigenetic mark and is important in both development and disease (1). It has been studied in numerous diseases to improve understanding of pathophysiology and to identify novel therapeutic targets and disease biomarkers (2). The advent of genome-wide DNAm arrays has enabled large, epigenome-wide association studies (EWAS). Specifically, the Illumina HumanMethylation450 (450k) and HumanMethylationEPIC (EPIC) BeadChips, which interrogate over 450,000 and 850,000 CpGs, respectively, have allowed researchers to interrogate human DNAm at an unprecedented scale.

The DNAm profile of each tissue and cell type is unique and can therefore differentiate cells from different organs (e.g., brain vs. lung), as well as different cell types within an organ (e.g., grey matter and white matter tissue in the brain have unique profiles) (3). As such, the biological sample used for EWAS should ideally be from a relevant organ/tissue (4). However, this is generally not possible in living humans. Furthermore, in samples which consist of multiple cell types, the outcome of interest may be confounded by differences in cell type composition (5). To address this, previous work has shown that the unique DNAm signature of individual cell types can be leveraged to estimate their relative proportions in mixed-cell samples such as blood (6 *).* These cell type proportions can then be accounted for in downstream statistical analysis (6). However, as was demonstrated by Bakulski et al (7), reference DNAm signatures of cell types from one source (e.g., adult blood) may not be perfectly representative of similar cell types from a different source (e.g., cord blood).

Clinical research of pulmonary diseases is currently limited by a lack of data from tissue and cell type specific samples. According to the EWAS Atlas, an online repository of published EWAS studies, there are 19 published EWAS studies in non-cancer lung diseases such as asthma, cystic fibrosis (CF), chronic obstructive pulmonary disease, acute respiratory distress syndrome and granulomatous lung disease (8). The majority have utilised samples from adult participants (13/19). Blood was the most commonly used biological sample in these studies (8/19 studies), followed by nasal and airway epithelial cells (6/19). The majority of these sample types do not contain immune cells, which are an integral part of the cellular milieu of the lung and are relevant to many lung diseases (9).

Bronchoalveolar lavage (BAL) is commonly collected as part of clinical care of lung disease. Particularly in young children, who cannot expectorate sputum, it is considered the gold-standard method of sampling the lung. BAL fluid permits assessment a variety of cells in the lung, including both circulating and resident immune cells, as well as respiratory epithelial cells (10). The predominant cell types in BAL are alveolar macrophages, granulocytes, lymphocytes and alveolar epithelial cells (AEC) (10–12). A current barrier to the widespread use of BAL samples in EWAS is the lack of a BAL-derived set of reference DNAm profiles for constituent cell types. Only three prior EWAS have utilised BAL samples, with varying approaches for adjustment of cell composition. One study, involving 8 participants (18-33years old), investigated only BAL macrophages, and used a reference-free approach to estimate and adjust for alveolar macrophage subtype composition (8). Another study, involving 48 adult participants, used microscopy derived lymphocyte cell counts to estimate the proportion of lymphocytes in the sample and adjust their analysis (13). This approach is limited by the resolution of microscopy and only accounts for lymphocytes in BAL, omitting other cell types (14, 15). A final study (16), involving 35 adult participants (median age 25.0, range: 22.0–29.0), used the publicly available eFORGE tool (17) to adjust for cell type composition. This tool was developed using a number of biological specimens such as fetal lung tissue and purified immune cell subsets. However, lung resident macrophages, which are the most common cell type in BAL, were not assessed. At present, there are no studies that have profiled the genome-wide DNAm of the cell types found in BAL, particularly from paediatric samples.

Here, we collected and purified individual cell populations from BAL collected from children with CF and generated cell-specific DNA methylation profiles with the aim of creating a BAL-derived DNAm reference panel of constituent cell types. Genome-wide DNAm profiles for 4 clinically-relevant BAL cell types (lymphocytes, granulocytes, alveolar macrophages, AECs) were measured using the Illumina EPIC array. We then used our BAL-derived reference panel in conjunction with a well-established deconvolution method to demonstrate its utility in the estimation of cell type proportions of BAL DNAm data. This BAL-specific reference panel will be useful for epigenetic studies of paediatric pulmonary disease.

## Materials and Methods

**Figure 1:**
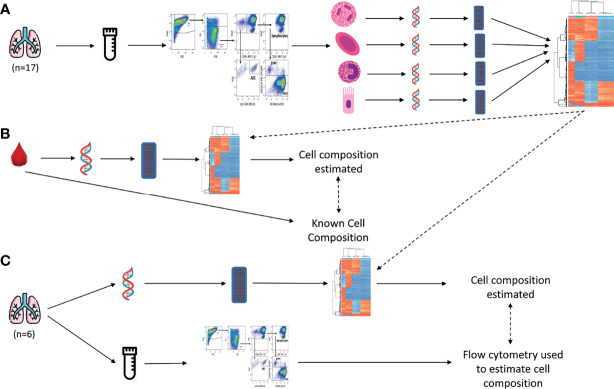
Study outline. **(A)** BAL samples were collected, cryopreserved and sorted using fluorescence-activated single cell sorting, resulting in 4 purified cell populations (Alveolar Epithelial Cells, Alveolar Macrophages, Granulocytes, and Lymphocytes). Their DNA was extracted and pooled and DNAm profiled on EPIC arrays. Unique DNAm profiles for constituent cell types were identified. These DNAm profiles were then used to estimate cell type compositions in subsequent validation experiments. **(B)** Data from a publicly available blood-derived artificial cell mixture with known cell composition, and matching DNAm data, was used to validate our BAL-derived reference panel. The BAL-derived DNAm profiles we developed were used in conjunction with a reference-based deconvolution method to estimate cell composition from the DNAm data of the artificial cell mixtures. This was then compared to the known cell composition. **(C)** BAL samples were collected and divided into a raw fraction and a cryopreserved fraction. DNA was extracted from the raw fraction, DNAm profiled, and our BAL-derived DNAm profiles used in conjunction with a reference-based deconvolution method to estimate cell composition. The cryopreserved fraction was profiled using flow cytometry, and cell composition estimated. The methylation-based estimates were compared to the flow-cytometry cell composition measurements.

A summary of the experimental workflow is shown in **Figure 1**. All analysis code presented in this manuscript can be found at https://jovmaksimovic.github.io/paed-BAL-meth-ref/index.html. The analysis website was created using the *workflowr* (1.6.2) R package (18). The GitHub repository associated with the analysis website is at: https://github.com/JovMaksimovic/paed-BAL-meth-ref.

### Sample Population and Biospecimen Collection

The 17 BAL samples used to establish the methylation reference panel of constituent cell types were obtained as part of the AREST CF study, with ethics approval (HREC #25054). We utilised excess BAL taken at the time of a clinically indicated procedures in children with CF (age 0 – 6 years). Flexible bronchoscopy was conducted by a respiratory physician under general anaesthesia. BAL was performed by instillation of sterile 0.9% normal saline in aliquots of one ml/kg (maximum 20ml). The aliquots used in this study were the second wash in the same bronchial tree location, which samples the distal airways this increasing the chances of sampling alveolar macrophages (19, 20). Samples were kept on ice after collection and cryopreserved within one hour, using previously described methods (10).

For estimation of constituent cell type proportions, BAL was collected from a further 6 subjects involved in the AREST CF study, as described above. Prior to processing, 1 mL of raw lavage was collected and stored at -80°C. The remaining BAL was cryopreserved as described. The cryopreserved fraction was then thawed and cell composition determined *via* flow cytometry using previously described methods (see “*Cell selection and Purification*” section for brief description) (10).

### Cell Selection and Purification

Cryopreserved samples were thawed and then fluorescence activated cell sorting was used to isolate alveolar macrophages, granulocytes, lymphocytes and AEC using previously described methods (10). Briefly the following markers were used to identify cell types: alveolar macrophages: CD45+, CD206+, granulocytes: CD45+, CD206-, CD15+, lymphocytes: CD45+, CD206-, CD15-, low forward scatter/side scatter, and AEC: CD45-, EpCAM+. Purified cell pellets were resuspended in 350uL of RLT buffer (Qiagen, Venlo, Netherlands) with 1% ß-mercaptoethanol (Gibco, New York, USA), and stored at -80°C until DNA extraction.

### DNA Extraction and Methylation Profiling

DNA was extracted from samples using the QIAamp DNA Micro Kit (Qiagen, Venlo, Netherlands). Aside from initial storage of the sample in RLT buffer with 1% ß-mercaptoethanol, the standard protocol for DNA extraction was used. DNA quantity and quality were assessed using a QUBIT<sp>® fluorometer and Nanodrop™ spectrophotometer respectively.

For purified cell type samples, the yield from an individual BAL was found to be insufficient for downstream whole genome methylation analysis. Thus, to avoid potential biases resulting from whole genome amplification (21, 22), DNA extracted from purified cell populations from the 17 individuals were pooled. This produced 5 macrophage pools (4 pools comprised of cells from 3 individuals, 1 comprised of cells from 2 individuals), 4 granulocyte pools (3 pools comprised of cells from 3 individuals, 1 pool comprised of cells from 5 individuals), 3 lymphocyte pools (2 pools comprised of cells from 6 individuals, 1 pool comprised of cells from 5 individuals) and 2 AEC pools (1 pool comprised of cells from 12 individuals, 1 pool comprised of cells from 5 individuals). To assess the impact of our pooling strategy, we examined the relationship between the number of individuals contributing to a pool and the variance across CpGs in the sample (**Supplementary Figure 1**). Regression analysis showed no statistically significant association between the number of individuals contributing to a pool and variance across CpGs (Adj. R^2^ = -0.02; p-value = 0.43), indicating that the sample pools were representative of the sorted cell types. Following extraction and pooling, DNA was stored at -30°C until further analysis.

The raw BAL samples, from 6 individuals, intended for cell composition estimation were thawed, and DNA was extracted and assessed for quality as previously described.

Genome-wide methylation profiling was performed for all samples using the Infinium MethylationEPIC array (EPIC array, Ilumina, San Diego, USA) at either GenomeScan (Leiden, Netherlands) or Erasmus Medical Centre (Rotterdam, Netherlands).

All of the data is available from the Gene Expression Omnibus: https://www.ncbi.nlm.nih.gov/geo/query/acc.cgi?acc=GSE185556.

### DNAm Data Pre-Processing

The EPIC array data were analysed using the R programming language (23) (Version 4.0.3) according to best practices for methylation array analysis (24). Raw IDAT files were imported using *minfi* (25, 26), followed by quality control. Firstly, as a measure of the quality of the array data, the detection p-value was calculated for each probe and any poor-quality samples with a mean detection p-value > 0.01 were excluded from subsequent analysis. The data was then normalised, using subset quantile normalisation (SQN) (27). Poor performing probes with detection p-value > 0.01 in one or more samples were excluded. In addition, probes known to have a single nucleotide polymorphism at the CpG site, probes that map to sex chromosomes, and cross-reactive probes that have been shown map to multiple places in the genome, were also excluded (28).

### Identification DNAm Cell Type Signature Probes

A modified version of the estimateCellCounts2 function from the *FlowSorted.Blood.EPIC* package (29), estimateCellCounts2Mod, was used to identify DNAm profiles unique to each cell type, and then estimate cell type proportions. This function implements the Houseman (6) deconvolution algorithm, but unlike the original *minfi* implementation, allows the use of a custom panel of reference epigenomes. Our estimateCellCounts2Mod function was specifically modified (https://github.com/JovMaksimovic/paed-BAL-meth-ref/blob/main/code/functions.R) to allow for the removal of probes excluded during quality control, prior to identification of DNAm cell type signature probes and cell proportion estimation.

We explored two *probeSelect* parameter options for DNAm cell type signature probe selection; *“both”* and *“any”*. Using *“both”*selects the top 50 hypermethylated and 50 hypomethylated probes (F-stat p-value < 1E-8) with the greatest methylation difference between each cell type compared to all the others. Using *“any”* selects the top 100 probes (F-stat p-value < 1E-8) with the greatest methylation difference between each cell type compared to all the others, regardless of direction of effect. The *processMethod* parameter, which determines how data will be normalised, was set to SQN (27).

Gene set enrichment analysis of probes selected using either the *“any”* or *“both”* options, and the probes that are in common and different between them was performed using the gometh function from the *missMethyl* (30) R Bioconductor package to account for known biases in gene set testing of methylation array data (31).

### Cell Type Proportion Estimation of Artificial Cell Mixtures

To assess the accuracy of cell proportions estimated using our BAL-derived reference panel in conjunction with the Houseman (6) algorithm, we utilised 12 publicly available artificial DNA mixtures profiled using Illumina Infinium HumanMethylationEPIC BeadChips (GSE110554). The mixtures were generated by combining known proportions of flow-sorted neutrophils, monocytes, B-lymphocytes, CD4+ T cells, CD8+ T cells, and natural killer cells (29). The data was downloaded using the *ExperimentHub* Bioconductor package (29). The known proportions of T, B and NK cells were summed to allow for comparison to the lymphocyte proportion estimated from the DNAm data using our BAL-derived reference panel, which only profiled total lymphocytes.

## Results

### Generation of a Methylation Reference Panel for BAL Derived Purified Cell Populations

Seventeen BAL samples obtained from children with CF were used for development of the BAL-specific reference panel. The median (range) age of the children was 36 months (14-70 months), and 11/17 (64.7%) were female. One child was of South-Asian ethnicity, and all other children were of European ethnicity. On average six mL of BAL was used. The median (range) cell composition of samples, determined by flow cytometry, was alveolar macrophages 63.7% (5.2%-95.9%), granulocytes 23.7% (2.9%-86.1%), lymphocytes 8.1% (0.7%-26.5%), and AEC 1.5% (0.2%-8.3%).

**Figure 2:**
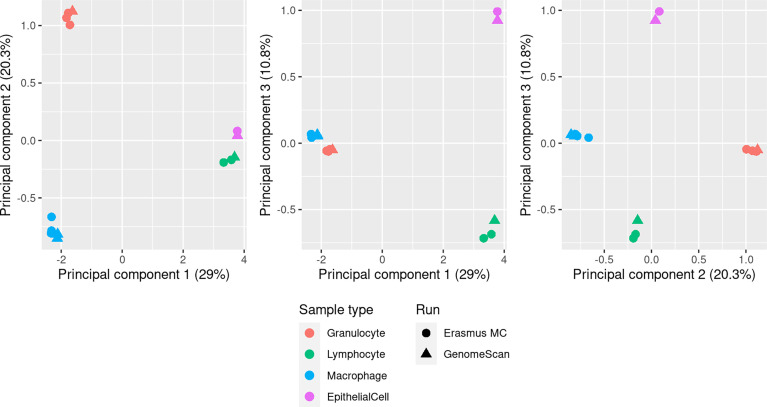
MDS plots showing the first 3 principal components of methylation data from pooled BAL macrophage (n=5), granulocyte (n=4), lymphocyte (n=3) and AEC (n=2) samples. Each pool contained purified cells from multiple individuals (see *Methods*). Clear separation of the different cell populations is seen in the first 3 principal components, which account for 60.1% of the total variation, and there is no evidence of a significant batch affect related to samples run at different service providers (Erasmus MC or GenomeScan).

DNAm of pooled purified BAL-derived macrophage, granulocyte, lymphocyte and AEC samples was profiled on EPIC arrays in 2 batches; 9 arrays were run at Erasmus MC and 11 arrays at GenomeScan. Following quality control, there were 732,778 probes remaining for further analysis. Multidimensional scaling (MDS) plots show strong clustering of the samples by cell type (**Figure 2**). A scree plot (**Supplementary Figure 2**) of the sources of variation in the data shows that the vast majority of the variation is explained by the first 4 principal components.

**Figure 3:**
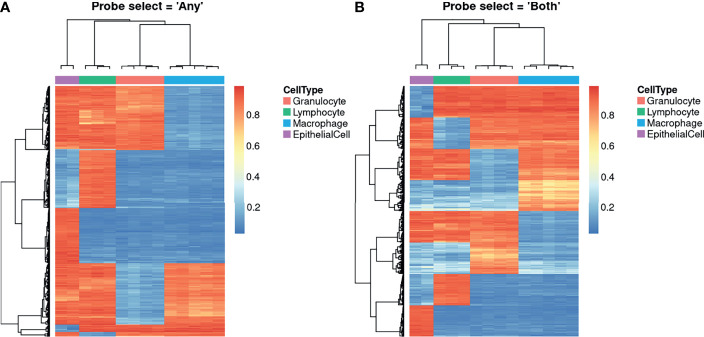
Heatmaps showing unique DNA methylation profiles for constituent cell types. The probes shown were algorithmically selected based on their ability to discriminate between the different cell types. The heatmaps demonstrate the different probe set selected when the *probeSelect* parameter is set to **(A)** *“any”* or **(B)** *“both”*. *“Both”* selects the top 50 hypermethylated and 50 hypomethylated probes (F-stat p-value < 1E-8) with the greatest methylation difference between each cell type compared to all the others. *“Any”* selects the top 100 probes (F-stat p-value < 1E-8) with the greatest methylation difference between each cell type compared to all the others, regardless of direction of effect.

The Houseman (6) deconvolution algorithm, as implemented in the *estimateCellCounts2* function from the *FlowSorted.Blood.EPIC* Bioconductor package (29), was used to identify DNA methylation profiles unique to each cell type. Briefly, the Houseman (6) algorithm is a type of regression calibration, originally developed using white blood cells, where a methylation pattern is considered to be a high-dimensional multivariate surrogate for the proportion of constituent cell in a sample of mixed cell types. It essentially leverages the DNAm profiles of purified cell types to estimate their relative proportions in a mixture. These estimates can subsequently be incorporated into statistical models to adjust for cell type composition in an EWAS (6, 32, 33), or independently investigated for their association with disease or environmental exposures (34–36). Cell composition was estimated with the *probeSelect* parameter set to “*both”* and then repeated with the “*any”* option. As shown in **Figure 3**, the probes selected using either option were able to clearly delineate the 4 cell types using hierarchical clustering. The selected probes are primarily associated with genes and overlap DNase hypersensitivity sites but most are not within CpG islands or in close proximity to the transcription start site (**Supplementary Figure 3**). Although there were no statistically significant terms at a false discovery rate less than 0.05, gene set enrichment analysis (GSEA) of probes selected using either option suggests that they are enriched for immune related-genes, which is unsurprising as ¾ of the contributing cell types were immune cells. Using the *“any”* option, the top 5 ranked Gene Ontology (GO) terms were “leukocyte activation”, “T cell activation”, “immune response” and “leukocyte cell-cell adhesion” (**Supplementary Table 1**). Similarly, using the *“both”* option, the top ranked GO terms were “leukocyte cell-cell adhesion”, “T cell activation”, leukocyte activation”, “lymphocyte activation” and “regulation of leukocyte cell-cell adhesion” (**Supplementary Table 2**). Of the 400 probes selected by each option, 221 (55.25%) were the same. GSEA of the overlapping probes also ranked various immune-related terms highly (**Supplementary Table 3**). The probes that differed between the *“any”* and *“both”* options also ranked some immune-specific terms highly, however, the top ranked terms were dominated by processes related to the JNK, JUN and MAPK cascades (**Supplementary Table 4**).

### Methylation-Based Cell Type Proportions Validated Using Artificial Cell Mixtures

**Figure 4:**
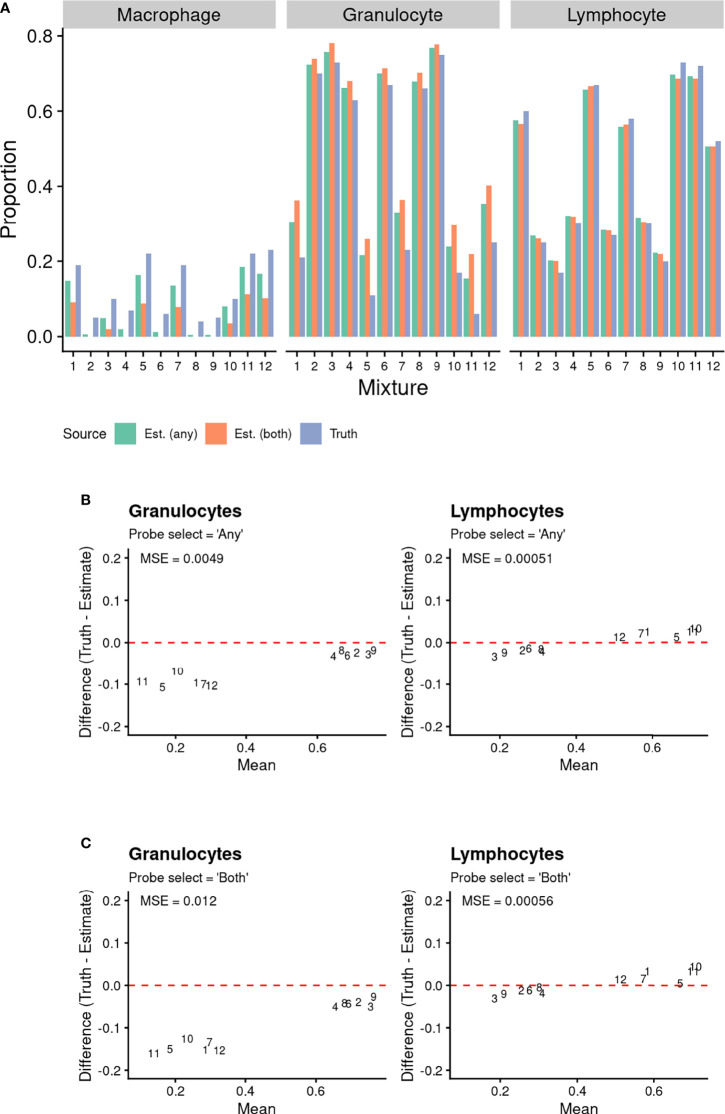
Comparison of known proportions of artificial cell mixtures (with T Cells, B Cells, and Natural Killer cells combined into a Lymphocyte population), to methylation-based estimated cell proportions derived using BAL purified cell population data. Cell type discriminating probes were selected using “any” and “both” approaches (see methods for details). **(A)** Comparison of known proportions of artificial cell mixtures (with T Cells, B Cells, and Natural Killer cells combined into a Lymphocyte population), to methylation-based estimated cell proportions derived using BAL purified cell population data. Cell type discriminating probes were selected using “any” and “both” approaches (see methods for details). **(B, C)** Bland Altman Plots Comparing Known Artificial Cell Mixture Proportions (“Truth”) and methylation-based cell proportion estimates (“Estimate”). The data point numbers represent which cell mixture the data pertains to. Cell type discriminating probes were selected using either **(B)** “any” and **(C)** “both” approaches (see *Methods* for details). The mean squared error (MSE) between the known proportion and estimated proportion was calculated for each cell type and probe selection *Method*.

We initially assessed cell proportion estimates underpinned by our BAL-specific cell reference panel using a publicly available dataset (29) of twelve artificially created cell mixtures. This allowed for validation of the accuracy of methylation-based estimates of BAL lymphocyte and granulocyte proportions. The results are summarised in **Figure 4**. For the cell types with available reference epigenomes (lymphocytes and granulocytes), there is a tight correlation between the true cell proportion and the methylation-based cell proportion estimated using our reference (**Figure 4A**). As expected, the sum of the estimated cell proportions was close to one (6). The methylation-based estimates include a proportion of alveolar macrophages not contained in the artificial cell mixture (**Figure 4A**). We expect that this is due to the presence of monocytes in the artificial cell mixture, which are macrophage precursor cells and are likely to share some similarity in their methylation patterns (37, 38). Furthermore, a fraction of alveolar macrophages is made up of circulating monocytes that migrate to the lungs and become a resident population (39, 40).

Overall, the mean squared error (MSE) (**Figures 4B, C**) between the true proportions and methylation-based estimates was close to zero for both lymphocytes and granulocytes, using either the “*any”* or “*both” probeSelect* option, indicating very high performance. However, the MSE for granulocytes was slightly higher than for lymphocytes, for both *probeSelect* options.

### Methylation-Based Cell Type Proportions in Paediatric BAL Samples

Six BAL samples were obtained for comparing cell type proportions determined by flow cytometry to methylation-based estimates generated using the Houseman method (6), in conjunction with our BAL-specific reference epigenomes. Five BALs were from children with CF, and one was from a “control” subject. Four of the six children (66.7%) were female. The median (range) age of the children was 57 months (23-89 months), which is slightly older than those used to derive the reference panel (median age 36 months, range 14-70 months). Five children were of European ethnicity and one was of South Asian ethnicity. All BAL samples had sufficient DNA extracted for methylation analysis.

The cell composition of all samples was determined using flow cytometry on cryopreserved BAL fractions (**Table 1**). For flow cytometry data, the proportion of cells identified ranged between 46.2% - 89.0%. The majority of the unidentified cells were CD45 –ve and EpCAM –ve indicating they were likely red blood cells. As red blood cells do not contain DNA, they do not contribute a DNA methylation signature. To support this, we identified 3479 CpG probes with mean ß values that were either ≥0.95 or ≤0.05 in all the sorted cell types, which should also be either fully methylated or unmethylated in the raw BAL samples if no other nucleated cell types are present. As shown in **Supplementary Figure 4**, these probes have consistent methylation levels across all the sorted cell types and the raw BAL samples, suggesting that cells not identified by flow cytometry were indeed red blood cells. Consequently, analysis was conducted including both the unknown cells (referred to as “original”) in the cell proportions and excluding them (referred to as “scaled”). The cell composition of most samples was as expected; however, two samples (CF1, CF5) had a CD45-,EpCAM- fraction in excess of 30% which is higher than expected and may relate to a traumatic blood-stained BAL.

**Table 1:** Cell composition of BAL determined by both DNA methylation-based estimate and flow cytometry.

	Alveolar Macrophage			Lymphocyte			Granulocyte			Alveolar Epithelial Cell		
	Scaled Flow Cytometry	DNAm Estimate (Any)	DNAm Estimate (Both)	Scaled Flow Cytometry	DNAm Estimate (Any)	DNAm Estimate (Both)	Scaled Flow Cytometry	DNAm Estimate (Any)	DNAm Estimate (Both)	Scaled Flow Cytometry	DNAm Estimate (Any)	DNAm Estimate (Both)
CF1	5.2	0.7	0.5	5.5	1.6	1.8	86.1	88.8	89.8	3.2	9.2	8.7
CF2	55.7	29.9	33.7	8.9	19.7	16.3	32.5	43.1	48.0	2.9	11.2	10.5
CF3	64.6	37.3	39.3	8	22.3	20.7	23.9	22.1	25.7	3.4	21.0	20.0
CF4	72.5	66.6	68.6	7.2	11.6	10.0	19.7	13.7	15.4	0.6	9.9	9.7
CF5	63.2	50.7	52.8	7.2	11.0	9.5	29.4	34.5	35.7	0.2	5.6	5.4
Control	51.1	13.7	13.1	20.5	17.8	16.7	21.5	58.7	61.7	6.9	12.9	12.8
** **	**Unscaled Flow Cytometry**	**DNAm Estimate (Any)**	**DNAm Estimate (Both)**	**Unscaled Flow Cytometry**	**DNAm Estimate (Any)**	**DNAm Estimate (Both)**	**Unscaled Flow Cytometry**	**DNAm Estimate (Any)**	**DNAm Estimate (Both)**	**Unscaled Flow Cytometry**	**DNAm Estimate (Any)**	**DNAm Estimate (Both)**
CF1	2.4	0.7	0.5	2.5	1.6	1.8	39.7	88.8	89.8	1.5	9.2	8.7
CF2	46.1	29.9	33.7	7.4	19.7	16.3	26.9	43.1	48.0	2.4	11.2	10.5
CF3	42.5	37.3	39.3	5.3	22.3	20.7	15.8	22.1	25.7	2.3	21.0	20.0
CF4	50.2	66.6	68.6	5	11.6	10.0	13.6	13.7	15.4	0.4	9.9	9.7
CF5	31.3	50.7	52.8	3.6	11.0	9.5	14.5	34.5	35.7	0.1	5.6	5.4
Control	34.8	13.7	13.1	13.9	17.8	16.7	14.6	58.7	61.7	4.7	12.9	12.8

For DNA methylation-based estimates cell type discriminating probes were selected using “Any” and “Both” approaches (see Methods for details). Regarding flow cytometry the “scaled” proportions were calculated not including the CD45-, EpCAM – cells which are likely Red Blood Cells that do not contribute to DNA methylation data.

**Figure 5:**
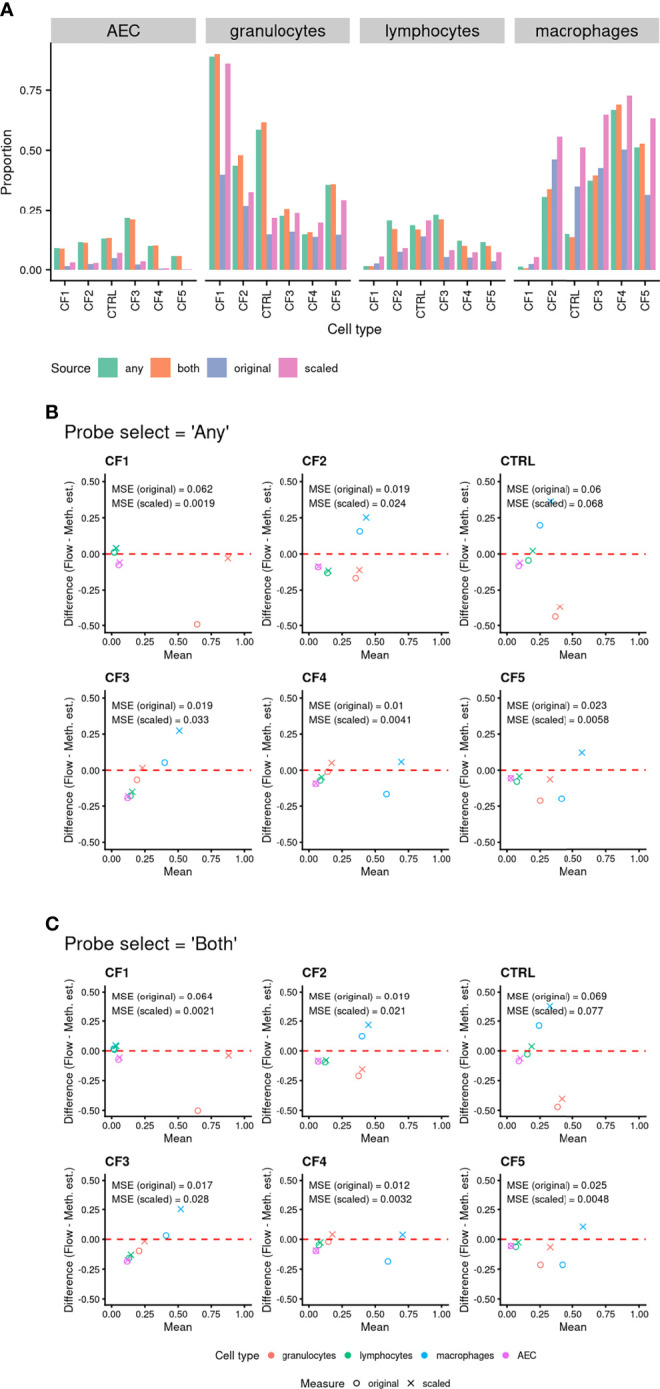
**(A)** Comparison of cell proportions measured *via* flow cytometry to methylation-based estimated cell proportions derived using BAL purified cell population data. Cell type discriminating probes were selected using *“any”* and *“both”* approaches (see methods for details). “Original” refers to the proportion of cells from flow cytometry in the total live cell population. “Scaled” refers to the proportion of cells when limited to just the four cell types of interest. **(B, C)** Bland Altman Plots Comparing cell proportions measured by flow cytometry (“Flow”) and methylation-based cell proportion estimates (“Meth. Est.”). The shapes indicate use of “original” (circle) or “scaled” (cross) flow cytometry proportions. The colour indicates the cell type (see legend). Probes were selected using either **(B)** *“any”* or **(C)** *“both”* approaches (see *Methods* for details). The mean squared error (MSE) between the known proportion and estimated proportion was calculated for each cell type and probe selection *Method*.

As for the artificial cell mixture data, methylation-based cell proportion estimates using the *“any”* or *“both”* probe selection methods were highly consistent across all cell types (**Figure 5A**). An MDS plot of the 6 BAL samples in the context of the purified cell type samples show them positioned centrally relative to the sorted cell samples (**Supplementary Figure 5**), with one BAL sample closer to the granulocyte cluster, suggesting a higher granulocyte proportion (**Supplementary Figure 5**). Cell proportions estimated using the methylation data and flow cytometry are compared in **Table 1** and **Figure 5**. The MSE between the “original” and “scaled” flow and methylation-based estimates was generally close to zero for all cell types and both *probeSelect* options, indicating reasonable concordance between the technologies (**Figures 5B, C**). The AEC and lymphocyte methylation-based estimates were the most concordant with the flow cytometry data. The alveolar macrophage and granulocyte proportions were more variable; in certain subjects (CF1 and control) there was a larger discrepancy between flow cytometry and DNAm estimated cell composition, reflected by a larger MSE of ~0.06, relative to the “original” flow estimates. Comparison to the “scaled” flow values markedly reduced the MSE for subjects CF1, CF4 and CF5 but had a negligible effect on the others.

## Discussion

We used the EPIC array to generate a reference panel of DNAm profiles for the four most common and clinically-relevant cell types in paediatric BAL: alveolar macrophages, granulocytes, lymphocytes and alveolar epithelial cells. The DNAm reference panel was then used to demonstrate estimation of cell composition of samples in two different datasets. Strengths of the work include purification of individual cell populations using FACS which was undertaken using previously validated, high-quality methods (10). The DNA methylation analysis and, in particular, the methods used for identifying cell-specific methylation probes have been well-established and widely used (6, 24, 29).

We compared known proportions of lymphocytes and granulocytes in an artificial cell mixture to estimates using DNAm with our BAL-specific reference panel in conjunction with the Houseman (6) deconvolution method. The known and estimated lymphocyte and granulocyte proportions were highly correlated, with MSE close to zero for cell types in all samples. In a subset of samples, the granulocyte proportion was slightly overestimated. This may be because the reference sample contains eosinophils as well as neutrophils and thus is therefore not a direct reflection of the cellular composition of the artificial cell mixture. Furthermore, in their original study, Houseman et al (6) also observed the most significant discrepancy between the true and estimated proportion for granulocytes.

We also estimated the cell type proportions of six paediatric BAL samples using our BAL-specific DNAm reference panel with the Houseman (6) deconvolution method and compared them to proportions measured using flow-cytometry. Two of the six subjects showed the greatest divergence between the flow-cytometry and DNAm estimates largely driven by differences between the granulocyte and alveolar macrophage proportions. The discrepancy in the granulocyte estimate is likely due to the effect of cryopreservation on the BAL fraction, which is known to reduce the proportion of granulocytes in a sample due to cell lysis (10). However, the BAL fraction profiled on EPIC arrays was not cryopreserved, thus retaining a higher proportion of granulocytes. The methylation-based estimate was consistently higher than that of flow cytometry strongly suggesting that the observed difference in proportions between flow-cytometry and DNAm granulocyte proportions is best explained by the effect of cryopreservation. The variable size of the discrepancy, may be due to the fact that cryopreservation primarily reduces the CD16+ granulocyte fraction (10). Thus, samples that originally had a large CD16+ granulocyte proportion are likely to have a larger discrepancy than those with a smaller CD16+ granulocyte proportion. We have previously shown the proportion of CD16+ granulocytes in BAL ranges from 0.09%-9% indicating substantial variation (10).

The variation in the methylation-based estimates of macrophages may be a consequence of the issues with the granulocyte estimate. The sum of the cell type proportions estimated by the Houseman method is expected to be close to one (6). Thus, if one cell proportion is inaccurately estimated there will be a reciprocal effect on other cell proportions. In this case, the consistent overestimation of granulocytes (when compared to flow cytometry data), may also result in the observed underestimation of the alveolar macrophage proportion. An alternate explanation could be heterogeneity of alveolar macrophage subpopulations, potentially related to disease severity. Recently, single cell transcriptomic analysis of adult BAL has revealed 13 alveolar macrophage subpopulations (41). The composition of a patient’s alveolar macrophage pool is likely related to disease severity, and each of the alveolar macrophage subpopulations will have a unique epigenetic profile. Thus, the discrepancy between the flow cytometry and methylation-based estimates of alveolar macrophage proportion seen in some subjects may be due to differences in the composition of their alveolar macrophage subtypes relative to the samples used to generate the reference epigenome. The relatively large discrepancy between the flow cytometry and methylation-based estimates of alveolar macrophage proportions for the control patient may reflect potentially altered alveolar macrophage DNA methylation profiles between control and CF patients (8).

It has been demonstrated that DNAm patterns related to exposure or disease can be confounded with differences in cell type proportions (32, 42). Furthermore, Bakulski et al (7) have shown that ensuring the DNAm reference panel used for cell proportion estimation is matched to the age of the study participants is especially important for some cell types. Adjusting using inaccurate cell proportion estimates may not completely resolve any confounding, underscoring the need to adjust for cell type proportions in BAL samples using an appropriate reference panel. The use of BAL in EWAS of pulmonary disease or exposure is appealing as it allows simultaneous assessment of methylation of local immune and epithelial cells, both of which are relevant to paediatric lung disease. Alternate biospecimens such as blood or bronchial brushings will omit cell types of interest. Our BAL-specific reference panel allows for deconvolution of multiple clinically-relevant cell types using any compatible reference-based method. While we used the Houseman method for estimating proportions, as this is a well-established and commonly used reference-based deconvolution method implemented in R, other methods could also be used in conjunction with the EPIC array data generated in this study. Furthermore, using a different probe selection strategy or varying F-statistic or beta value thresholds However, given the reference panel was derived from children with CF, it may need further validation prior to use on samples from different disease groups and ages. Although, its excellent performance when used to estimate the composition of artificial cell mixtures derived from healthy adult blood, does support that the reference panel will be suitable for broader populations.

We expect that this novel BAL-specific sorted cell type DNAm reference panel will be widely utilised by the paediatric pulmonary research community for facilitating EWAS of paediatric pulmonary diseases. Based on our findings, we would recommend the use of this reference panel on genomic DNA extracted from freshly isolated BAL samples.

## Data Availability Statement

The original contributions presented in the study are publicly available. This data can be found here: https://www.ncbi.nlm.nih.gov/geo/query/acc.cgi?acc=GSE185556.

## Ethics Statement

The studies involving human participants were reviewed and approved by Royal Children’s Hospital. Written informed consent to participate in this study was provided by the participants’ legal guardian/next of kin.

## Author Contributions

All authors contributed to the design of the study. SS and SR were responsible for recruitment of participants and collection of bronchoalveolar lavage samples. SS and MN were responsible for cryopreservation and flow cytometry. SS was responsible for DNA extraction and arranging DNA methylation analysis. JM was primarily responsible for the analysis of the data with support from SS, RS and AO. All authors contributed to interpretation of the findings. SS and JM were primarily responsible for drafting the manuscript with MN, SR, AO, and RS involved in editing and revision. All authors contributed to the article and approved the submitted version.

## Funding

This work was funded by a Vertex Pharmaceuticals Research Innovation Award (Ranganathan 2017).

## Conflict of Interest

The authors declare that the research was conducted in the absence of any commercial or financial relationships that could be construed as a potential conflict of interest.

## Publisher’s Note

All claims expressed in this article are solely those of the authors and do not necessarily represent those of their affiliated organizations, or those of the publisher, the editors and the reviewers. Any product that may be evaluated in this article, or claim that may be made by its manufacturer, is not guaranteed or endorsed by the publisher.
