# Supplementary material for: DNA Methylation Profiles of Purified Cell Types in Bronchoalveolar Lavage: Applications for Mixed Cell Paediatric Pulmonary Studies
Source: Front Immunol. 2021 Dec 22;12:788705. doi: 10.3389/fimmu.2021.788705 (PMC8727592; doi:10.3389/fimmu.2021.788705)
Supplement: Supplementary file 1 [file DataSheet_1.docx]

Supplementary Material

# Supplementary Figures and Tables

## Supplementary Figures


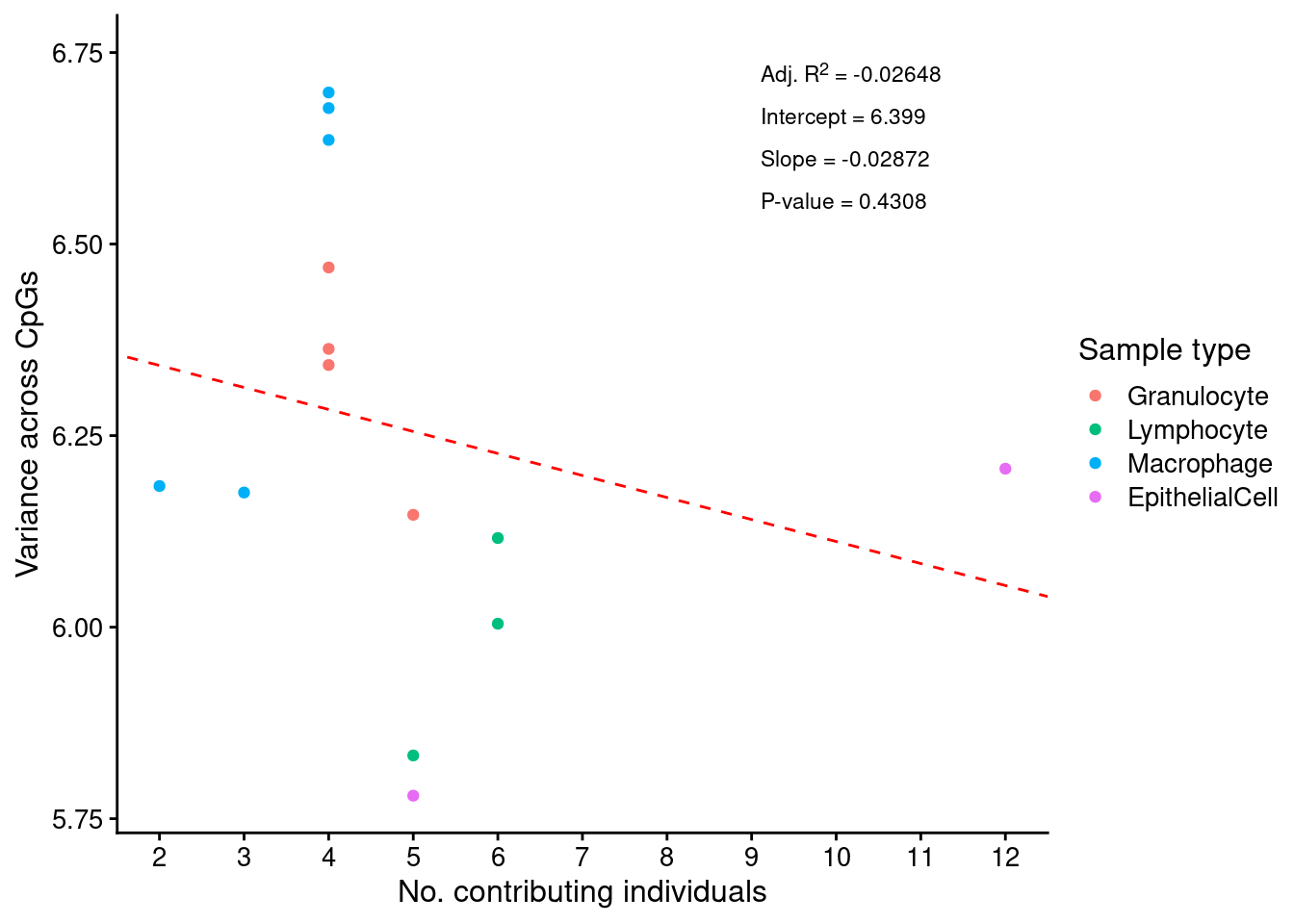


**Supplementary Figure 1**. The relationship between the number of individuals contributing to a pooled sorted cell type sample and the variance across CpGs in each corresponding sample. A linear regression analysis found no statistically significant association between the number of individuals contributing to a pool and its variance across CpGs.


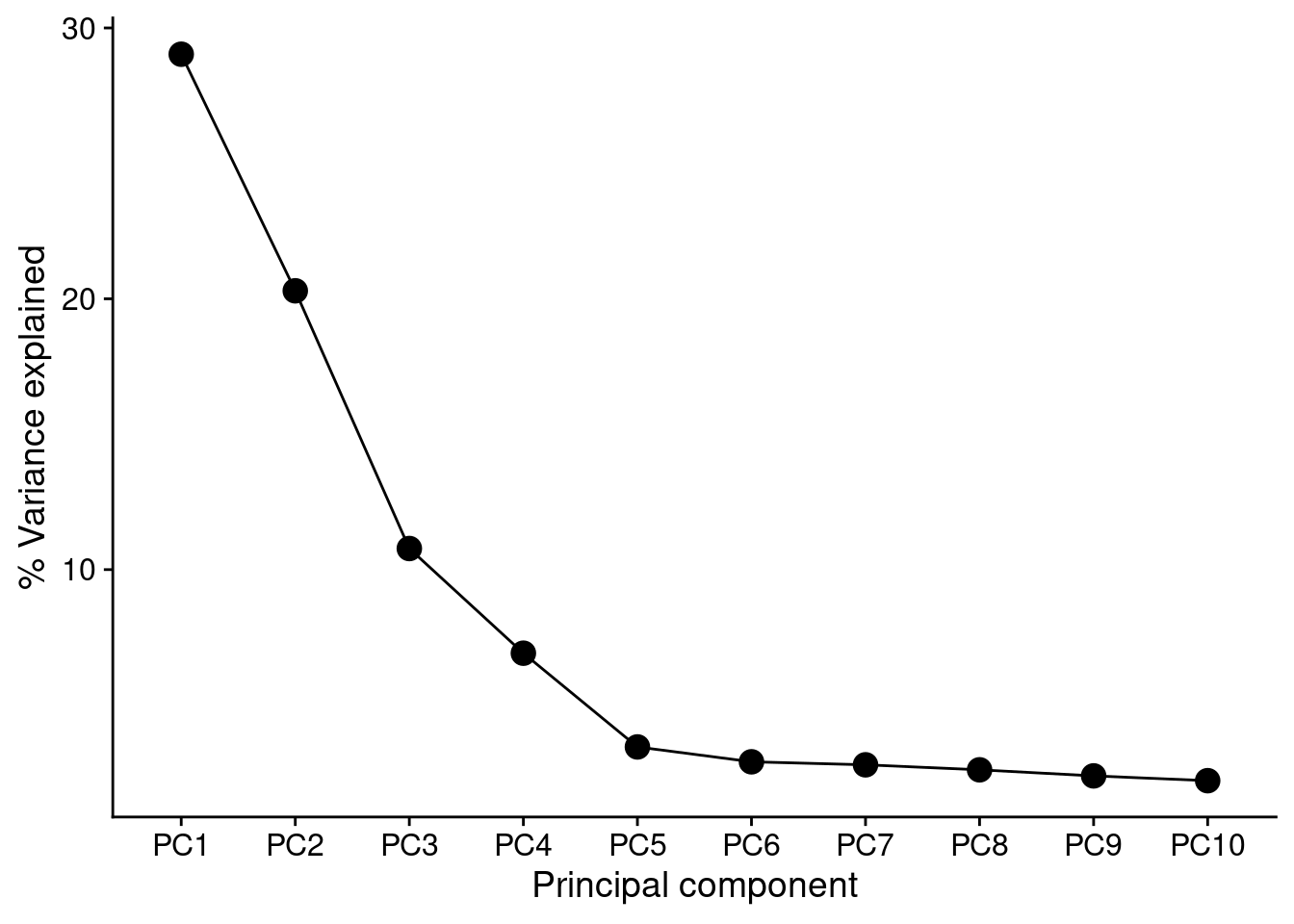


**Supplementary Figure 2.** Scree plot showing the percentage of variation explained by each of the top 10 principal components. The majority of the variation in this data can be explained by the top 4 principal components.


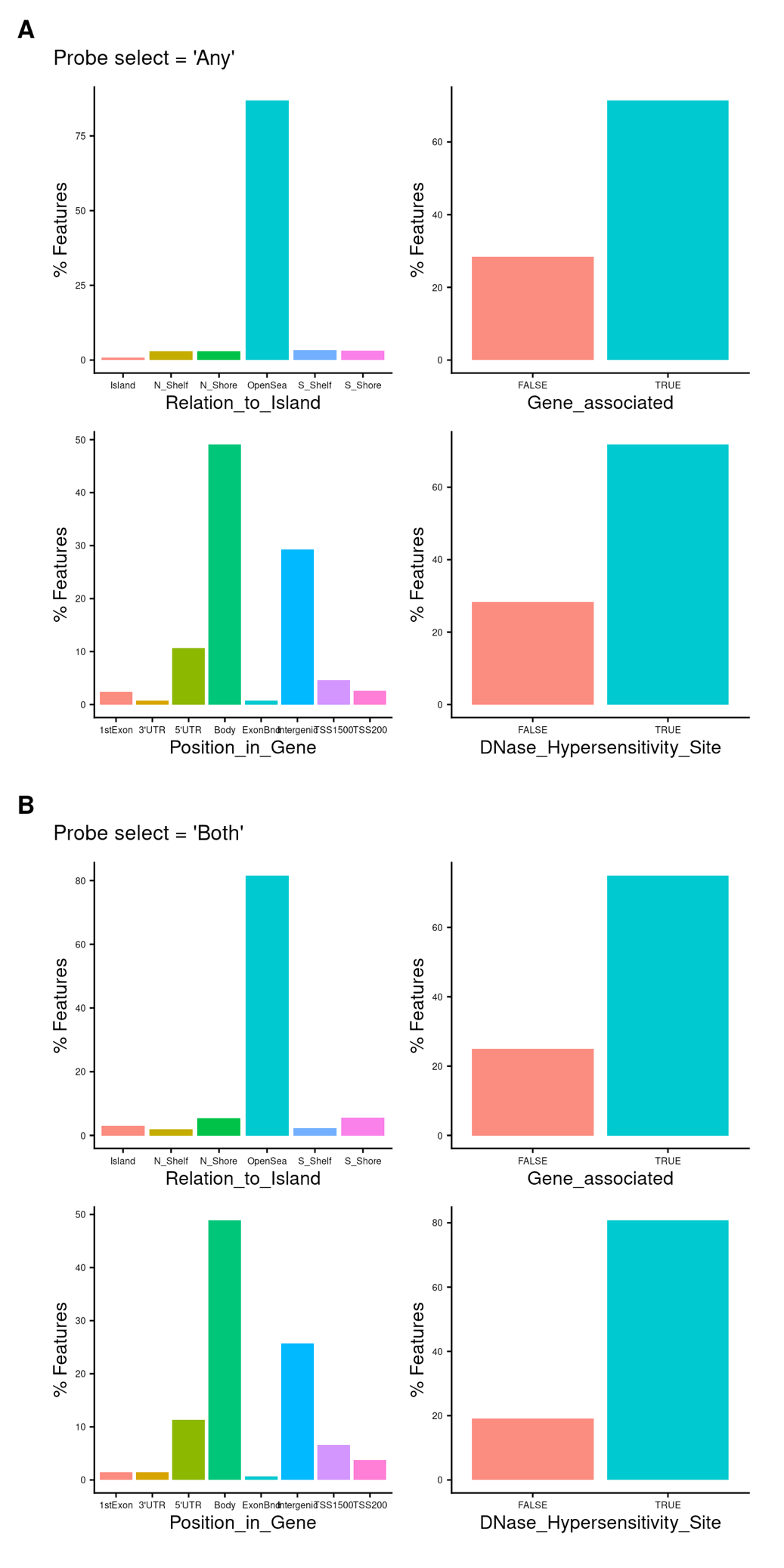


**Supplementary Figure 3.** Summary of the genomic context of the cell discriminating probes selected using either the (A) *“any”* or (B) *“both”* *probeSelect* options. The probes selected by either option tend to be associated with genes and often overlap with known DNase hypersensitivity sites but are mostly not in CpG islands or in close proximity to the transcription start site.


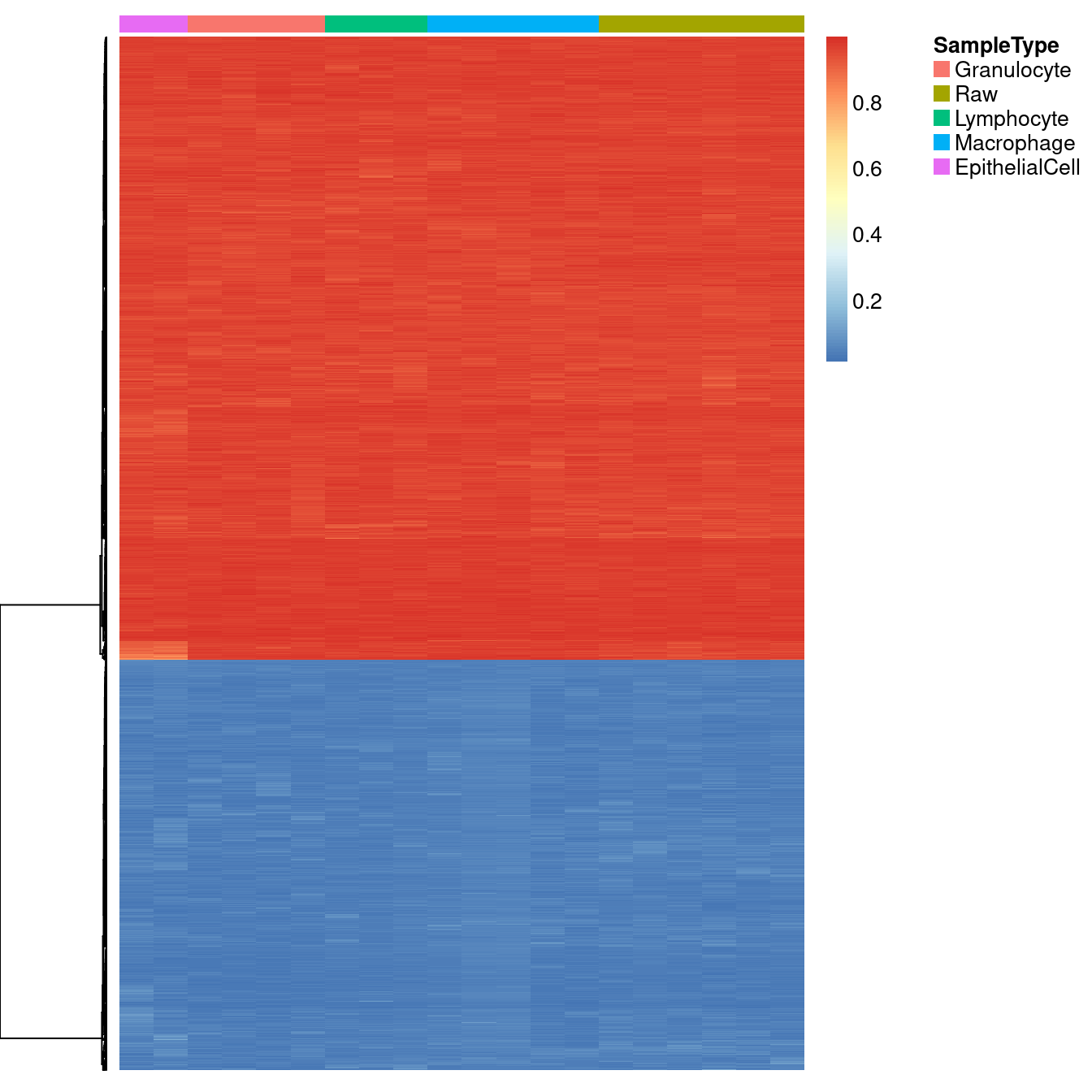


**Supplementary Figure 4.** Heatmap showing 3479 probes with mean β values that are either greater than 0.95 or less than 0.05 across all the sorted cell types. These probes are also consistently fully methylated or fully unmethylated in the raw BAL samples, confirming that the raw BAL is unlikely to contain additional nucleated cell types.


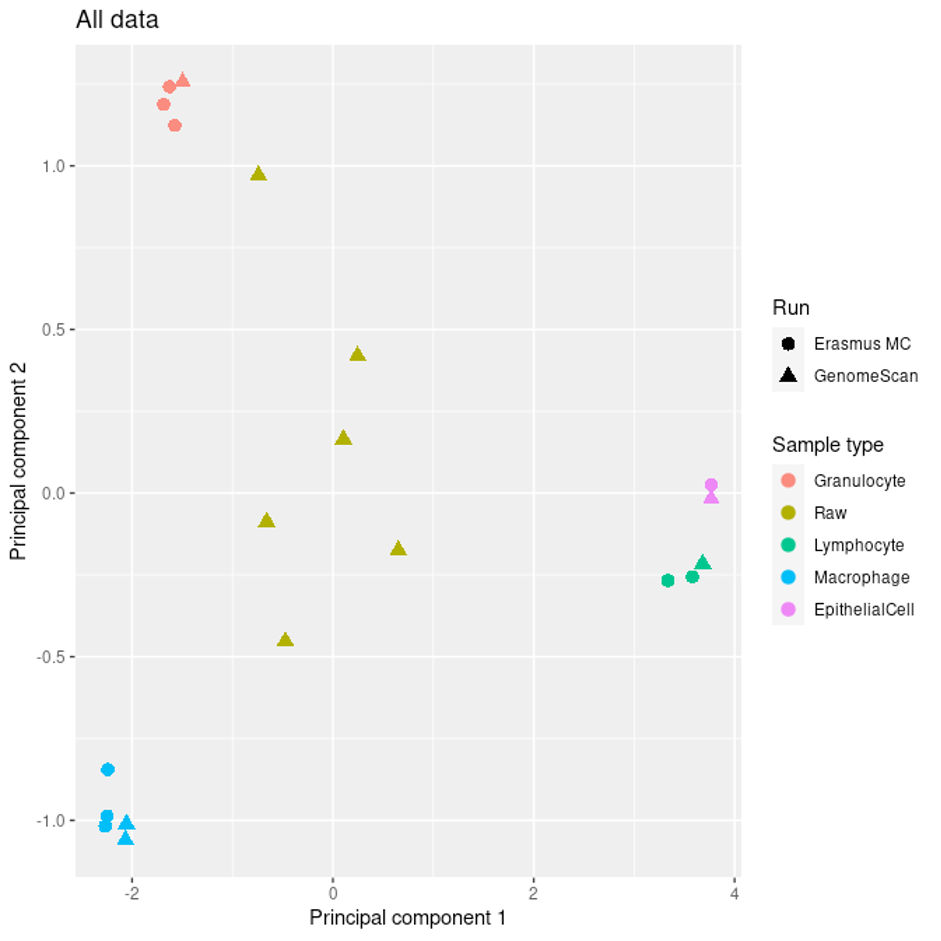


**Supplementary Figure 5.** MDS plot showing that the 6 BAL samples (labelled RAW) sit centrally relative to the sorted cell samples, with one BAL sample closer the granulocyte cluster, suggesting a higher granulocyte proportion

## Supplementary Tables

**Supplementary Table 1.** Top 20 ranked GO terms from gene set enrichment analysis of probes selected using the *“any”* option.

| GO ID | ONTOLOGY | TERM | N | DE | P.DE | FDR |
| --- | --- | --- | --- | --- | --- | --- |
| <GO:0045321> | BP | leukocyte activation | 1197 | 37 | 0.0000054 | 0.1223846 |
| <GO:0042110> | BP | T cell activation | 464 | 20 | 0.0000268 | 0.2365053 |
| <GO:0006955> | BP | immune response | 1912 | 45 | 0.0000431 | 0.2365053 |
| <GO:0007159> | BP | leukocyte cell-cell adhesion | 348 | 16 | 0.0000447 | 0.2365053 |
| <GO:1903037> | BP | regulation of leukocyte cell-cell adhesion | 314 | 15 | 0.0000523 | 0.2365053 |
| <GO:0001775> | BP | cell activation | 1351 | 38 | 0.0000648 | 0.2442241 |
| <GO:0050851> | BP | antigen receptor-mediated signaling pathway | 235 | 13 | 0.0001199 | 0.3725532 |
| <GO:0002684> | BP | positive regulation of immune system process | 950 | 28 | 0.0001597 | 0.3725532 |
| <GO:0002768> | BP | immune response-regulating cell surface receptor signaling pathway | 384 | 17 | 0.0001673 | 0.3725532 |
| <GO:0002764> | BP | immune response-regulating signaling pathway | 387 | 17 | 0.0001731 | 0.3725532 |
| <GO:0002429> | BP | immune response-activating cell surface receptor signaling pathway | 353 | 16 | 0.0001976 | 0.3725532 |
| <GO:0002757> | BP | immune response-activating signal transduction | 353 | 16 | 0.0001976 | 0.3725532 |
| <GO:0002682> | BP | regulation of immune system process | 1458 | 37 | 0.0002646 | 0.4604714 |
| <GO:0046649> | BP | lymphocyte activation | 661 | 22 | 0.0003418 | 0.5270029 |
| <GO:0042060> | BP | wound healing | 514 | 21 | 0.0003495 | 0.5270029 |
| <GO:0050900> | BP | leukocyte migration | 442 | 16 | 0.0005924 | 0.7996714 |
| <GO:0045579> | BP | positive regulation of B cell differentiation | 11 | 3 | 0.0006356 | 0.7996714 |
| <GO:0042117> | BP | monocyte activation | 10 | 3 | 0.0006594 | 0.7996714 |
| <GO:0002252> | BP | immune effector process | 1149 | 29 | 0.0006717 | 0.7996714 |
| <GO:0002280> | BP | monocyte activation involved in immune response | 2 | 2 | 0.0007406 | 0.8376712 |

**Supplementary Table 2.** Top 20 ranked GO terms from gene set enrichment analysis of probes selected using the *“both”* option.

| GO ID | ONTOLOGY | TERM | N | DE | P.DE | FDR |
| --- | --- | --- | --- | --- | --- | --- |
| <GO:0007159> | BP | leukocyte cell-cell adhesion | 348 | 17 | 0.0000247 | 0.5583832 |
| <GO:0042110> | BP | T cell activation | 464 | 19 | 0.0001745 | 1.0000000 |
| <GO:0045321> | BP | leukocyte activation | 1197 | 34 | 0.0002183 | 1.0000000 |
| <GO:0046649> | BP | lymphocyte activation | 661 | 23 | 0.0002950 | 1.0000000 |
| <GO:1903037> | BP | regulation of leukocyte cell-cell adhesion | 314 | 14 | 0.0003422 | 1.0000000 |
| <GO:0006955> | BP | immune response | 1912 | 44 | 0.0003422 | 1.0000000 |
| <GO:0001775> | BP | cell activation | 1351 | 37 | 0.0004153 | 1.0000000 |
| <GO:0044272> | BP | sulfur compound biosynthetic process | 181 | 10 | 0.0005641 | 1.0000000 |
| <GO:0042117> | BP | monocyte activation | 10 | 3 | 0.0007333 | 1.0000000 |
| <GO:0002280> | BP | monocyte activation involved in immune response | 2 | 2 | 0.0007949 | 1.0000000 |
| <GO:0002682> | BP | regulation of immune system process | 1458 | 37 | 0.0007958 | 1.0000000 |
| <GO:0002684> | BP | positive regulation of immune system process | 950 | 27 | 0.0008903 | 1.0000000 |
| <GO:0036037> | BP | CD8-positive, alpha-beta T cell activation | 27 | 4 | 0.0010062 | 1.0000000 |
| <GO:1903039> | BP | positive regulation of leukocyte cell-cell adhesion | 225 | 11 | 0.0010420 | 1.0000000 |
| <GO:0048010> | BP | vascular endothelial growth factor receptor signaling pathway | 95 | 8 | 0.0010930 | 1.0000000 |
| <GO:0050776> | BP | regulation of immune response | 868 | 24 | 0.0011057 | 1.0000000 |
| <GO:0033292> | BP | T-tubule organization | 9 | 3 | 0.0012279 | 1.0000000 |
| <GO:0000506> | CC | glycosylphosphatidylinositol-N-acetylglucosaminyltransferase (GPI-GnT) complex | 7 | 2 | 0.0012681 | 1.0000000 |
| <GO:0007256> | BP | activation of JNKK activity | 10 | 3 | 0.0012843 | 1.0000000 |
| <GO:0015961> | BP | diadenosine polyphosphate catabolic process | 4 | 2 | 0.0013242 | 1.0000000 |

**Supplementary Table 3.** Top 20 ranked GO terms from gene set enrichment analysis of probes that are the same using the *“any”* and *“both”* options.

| GO ID | ONTOLOGY | TERM | N | DE | P.DE | FDR |
| --- | --- | --- | --- | --- | --- | --- |
| <GO:0042117> | BP | monocyte activation | 10 | 3 | 0.0001583 | 1 |
| <GO:0033292> | BP | T-tubule organization | 9 | 3 | 0.0002727 | 1 |
| <GO:0002280> | BP | monocyte activation involved in immune response | 2 | 2 | 0.0002863 | 1 |
| <GO:0007159> | BP | leukocyte cell-cell adhesion | 348 | 11 | 0.0002880 | 1 |
| <GO:0000506> | CC | glycosylphosphatidylinositol-N-acetylglucosaminyltransferase (GPI-GnT) complex | 7 | 2 | 0.0004144 | 1 |
| <GO:0050765> | BP | negative regulation of phagocytosis | 21 | 3 | 0.0005205 | 1 |
| <GO:1903037> | BP | regulation of leukocyte cell-cell adhesion | 314 | 10 | 0.0005293 | 1 |
| <GO:0047756> | MF | chondroitin 4-sulfotransferase activity | 3 | 2 | 0.0005562 | 1 |
| <GO:0097470> | CC | ribbon synapse | 11 | 3 | 0.0006711 | 1 |
| <GO:0010452> | BP | histone H3-K36 methylation | 14 | 3 | 0.0006845 | 1 |
| <GO:0090023> | BP | positive regulation of neutrophil chemotaxis | 25 | 3 | 0.0010073 | 1 |
| <GO:0010793> | BP | regulation of mRNA export from nucleus | 5 | 2 | 0.0011003 | 1 |
| <GO:0071624> | BP | positive regulation of granulocyte chemotaxis | 28 | 3 | 0.0011239 | 1 |
| <GO:0034481> | MF | chondroitin sulfotransferase activity | 4 | 2 | 0.0011489 | 1 |
| <GO:2000197> | BP | regulation of ribonucleoprotein complex localization | 6 | 2 | 0.0013055 | 1 |
| <GO:1903039> | BP | positive regulation of leukocyte cell-cell adhesion | 225 | 8 | 0.0013234 | 1 |
| <GO:1902624> | BP | positive regulation of neutrophil migration | 27 | 3 | 0.0014916 | 1 |
| <GO:0043378> | BP | positive regulation of CD8-positive, alpha-beta T cell differentiation | 4 | 2 | 0.0014967 | 1 |
| <GO:0022409> | BP | positive regulation of cell-cell adhesion | 269 | 9 | 0.0015263 | 1 |
| <GO:0042060> | BP | wound healing | 514 | 14 | 0.0016411 | 1 |

**Supplementary Table 4.** Top 20 ranked GO terms from gene set enrichment analysis of probes that are different between the *“any”* and *“both”* options.

| GO ID | ONTOLOGY | TERM | N | DE | P.DE | FDR |
| --- | --- | --- | --- | --- | --- | --- |
| <GO:0004674> | MF | protein serine/threonine kinase activity | 412 | 21 | 0.0000145 | 0.2751161 |
| <GO:0006955> | BP | immune response | 1912 | 41 | 0.0000243 | 0.2751161 |
| <GO:0046328> | BP | regulation of JNK cascade | 177 | 12 | 0.0000507 | 0.2842184 |
| <GO:0018210> | BP | peptidyl-threonine modification | 123 | 10 | 0.0000672 | 0.2842184 |
| <GO:0046330> | BP | positive regulation of JNK cascade | 131 | 10 | 0.0000751 | 0.2842184 |
| <GO:0043506> | BP | regulation of JUN kinase activity | 85 | 8 | 0.0000871 | 0.2842184 |
| <GO:0046649> | BP | lymphocyte activation | 661 | 21 | 0.0001081 | 0.2842184 |
| <GO:0048584> | BP | positive regulation of response to stimulus | 2258 | 51 | 0.0001149 | 0.2842184 |
| <GO:0045321> | BP | leukocyte activation | 1197 | 30 | 0.0001177 | 0.2842184 |
| <GO:0007254> | BP | JNK cascade | 201 | 12 | 0.0001341 | 0.2842184 |
| <GO:0080135> | BP | regulation of cellular response to stress | 740 | 24 | 0.0001382 | 0.2842184 |
| <GO:0018107> | BP | peptidyl-threonine phosphorylation | 114 | 9 | 0.0001825 | 0.3088682 |
| <GO:0032874> | BP | positive regulation of stress-activated MAPK cascade | 162 | 10 | 0.0002110 | 0.3088682 |
| <GO:0018209> | BP | peptidyl-serine modification | 316 | 15 | 0.0002165 | 0.3088682 |
| <GO:0043507> | BP | positive regulation of JUN kinase activity | 70 | 7 | 0.0002184 | 0.3088682 |
| <GO:0034097> | BP | response to cytokine | 1166 | 28 | 0.0002292 | 0.3088682 |
| <GO:0032872> | BP | regulation of stress-activated MAPK cascade | 226 | 12 | 0.0002321 | 0.3088682 |
| <GO:0070304> | BP | positive regulation of stress-activated protein kinase signaling cascade | 164 | 10 | 0.0002533 | 0.3151636 |
| <GO:0034351> | BP | negative regulation of glial cell apoptotic process | 7 | 3 | 0.0002750 | 0.3151636 |
| <GO:0070302> | BP | regulation of stress-activated protein kinase signaling cascade | 229 | 12 | 0.0002786 | 0.3151636 |
